# Thymoquinone affects the gemcitabine sensitivity of pancreatic cancer by regulating collagen via hypoxia inducible factor-1α

**DOI:** 10.3389/fphar.2023.1138265

**Published:** 2023-05-31

**Authors:** Zhanxue Zhao, Linxun Liu, Hekai Chen, Shuai Li, Yan Guo, Xiaofan Hou, Jinyu Yang

**Affiliations:** ^1^ Suzhou Medical College of Soochow University, Suzhou, Jiangsu, China; ^2^ Department of General Surgery, Qinghai Provincial People’s Hospital, Xining, Qinghai, China; ^3^ Department of General Surgery, Peking University BinHai Hospital, Tianjin, China; ^4^ Department of Clinical Pharmacy, Affiliated Hospital of Qinghai University, Xining, Qinghai, China; ^5^ Department of Pathology, Qinghai Provincial People’s Hospital, Xining, Qinghai, China; ^6^ Graduate School, Qinghai University, Xining, Qinghai, China

**Keywords:** thymoquinone, pancreatic cancer, HIF-1α, ECM production, gemcitabine

## Abstract

**Objective:** To clarify the potential therapeutic effects of thymoquinone (TQ) on pancreatic cancer and its gemcitabine (GEM) sensitivity.

**Methods:** The expression levels of hypoxia inducible factor-1α (HIF-1α), collagens (COL1A1, COL3A1, and COL5A1), and transforming growth factor-β1 (TGFβ1) in pancreatic cancer and para-carcinoma tissues were compared using immunohistochemical methods, and their relationships with TNM staging were analyzed. The effects of TQ on apoptosis, migration, invasion, and GEM sensitivity of pancreatic cancer cells were assessed using *in vitro* and *in vivo* experiments. Western blot and immunohistochemistry were used to detect the expression levels of HIF-1α, extracellular matrix (ECM) production pathway-related proteins, and TGFβ/Smad signaling pathway-related proteins.

**Results:** The expression levels of HIF-1α, COL1A1, COL3A1, COL5A1, and TGFβ1 in pancreatic cancer tissues were significantly higher than those in para-carcinoma tissues and correlated with TNM staging (*p* < 0.05). TQ and GEM administration inhibited the migration and invasion of the human pancreatic cancer cell line PANC-1 and promoted the apoptosis of PANC-1 cells. The combination of TQ and GEM was more effective than GEM alone. Western blot analysis showed that the expression levels of HIF-1α, ECM production pathway-related proteins, and TGFβ/Smad signaling pathway-related proteins were significantly decreased when TQ was used to treat PANC-1 cells (*p* < 0.05), and the expression levels of these proteins in the TQ + GEM group were significantly more decreased than those in the GEM group. Overexpression or knockdown of HIF-1α in PANC-1 cells showed the same effects as those induced by TQ administration. *In vivo* experiments showed that in PANC-1 tumor-bearing mice, tumor volume and tumor weight in mice treated with GEM and TQ were significantly lower than those in control or GEM-treated mice, whereas cell apoptosis was significantly increased (*p* < 0.05). Western blot and immunohistochemistry results showed that the levels of HIF-1α, ECM production pathway-related proteins, and TGFβ/Smad signaling pathway-related proteins in the GEM + TQ treatment group were further decreased compared to the control group or the GEM treatment group (*p* < 0.05).

**Conclusion:** In pancreatic cancer cells, TQ can promote apoptosis, inhibit migration, invasion, and metastasis, and enhance the sensitivity to GEM. The underlying mechanism may involve the regulation of ECM production through the TGFβ/Smad pathway, in which HIF-1α plays a key role.

## 1 Introduction

Pancreatic cancer (PC) is one of the most malignant tumors of the digestive system. According to the global cancer statistics of 2018, PC accounts for 2.5% of new cancers, and the mortality rate accounts for 4.5% of all cancer-related deaths (Bray et al., 2018). Its incidence and mortality have increased significantly in recent years because of dietary, hereditary, environmental, and other factors (Goral, 2015; Siegel et al., 2021). The main difficulty in the diagnosis and treatment of PC lies in its occult onset and rapid metastasis (Chu et al., 2017; Tempero, 2019). The high metastasis rate, low chemosensitivity, and high mortality rate of PC are also poorly understood. At present, the clinical treatment for PC mainly includes surgery, radiotherapy, chemotherapy, interventional therapy, and supportive treatment. More than 80% of patients with PC not only lose the opportunity of surgery due to the late discovery of the disease, but the clinical implementation of a comprehensive treatment based on radiotherapy and chemotherapy is also not ideal.

Hypoxia inducible factor-1 (HIF-1) was first reported in 1992. It is an oxygen-dependent transcription factor and a key factor in regulating cell adaptation to hypoxia (Freedman et al., 2002). In addition, Li et al. discovered that HIF-1α, combining with chemotactic factor 2 (CCL2), recruits macrophages to activate pancreatic stellate cells, promoting the secretion of extracellular matrix (ECM) in PC, aggravating hypoxia of the pancreatic microenvironment, and promoting the progression of PC (Li et al., 2016). Other studies found that HIF-1α increases the expression of Sonic hedgehog protein (SHH) in a HIF-1α-dependent manner, and its overexpression activates hedgehog signal transduction and the formation of collagen I and fibronectin, enhancing the invasion of PC (Spivak-Kroizman et al., 2013; Katagiri et al., 2018).

Black seed oil, which is derived from the *Nigella sativa* plant, is a traditional medicinal product used in Arab countries, South and Southeast Asia, the Mediterranean, China, and various African countries. The components of black seeds include fixed oil (22%–38%), volatile oil (0.40%–0.45%), alkaloids (0.01%), amino acids and proteins (21%–31%), carbohydrates (25%–40%), saponins (0.013%), vitamins (1%–4%), minerals (3.7%–7%), and terpenoids, p-isocyanate, limonene, thiamine, niacin, and folic acid with different compositions (Shafodino et al., 2022). The main fatty acids of fixed oil are linoleic acid (64.6%) and palmitic acid (20.4%), whereas the identified amino acids mainly comprise cysteine, methionine, glutamic acid, aspartic acid, and arginine. The minerals mainly include iron, copper, zinc, phosphorus, and calcium (Yimer et al., 2019). Moreover, volatile black seed oils contain approximately 18.4%–24.0% of thymoquinone (TQ) as the main bioactive ingredient. TQ exhibits a wide range of pharmacological activities, including antioxidant, anti-inflammatory, antifibrotic, antidiabetic, antihistamine, anticancer, antimicrobial, and anticonvulsive effects (Mansour et al., 2001; Kanter, 2011; Goyal et al., 2017). Regarding anticancer properties, previous studies have shown that TQ participates in the regulation of tumor cell proliferation, apoptosis, metastasis, and drug resistance, among others (Imran et al., 2018; Alhmied et al., 2021; Malik et al., 2021).

Tumor heterogeneity, interstitial fibrosis, and an immunosuppressive microenvironment are the main reasons for the high mortality in patients with PC. Among these factors, interstitial fibrosis and hypoxia are the main characteristics that distinguish PC from other cancers (Zhuo et al., 2018). Pancreatic stellate cells and cancer-related fibroblasts in the microenvironment of PC tumors secrete a large amount of collagen and other components through a variety of pathways, forming a fibrotic barrier. At the same time, the physiological activities of cancer cells promote the development of interstitial fibrosis. Interstitial fibrosis in the PC microenvironment is considered the main reason for the low efficacy of chemotherapeutic drug delivery. Degradation of collagen accumulation in interstitial fibrosis helps to improve drug delivery and perfusion (Provenzano et al., 2012). Collagen can not only inhibit the effects of chemotherapy drugs by acting as a physical barrier but also enhances the drug resistance of cancer cells to gemcitabine (GEM) through multiple pathways (Shields et al., 2012; Dangi-Garimella et al., 2013). A prior study also found that collagen fibers cannot block the migration of tumor cells but act instead as a “highway” to spread and transfer cancer cells (Hamada and Masamune, 2018) and promote the epithelial-mesenchymal transition of PC (Duan et al., 2014; Huang et al., 2016). There are dozens of collagen proteins, but the main collagen proteins in PC are collagen I, III, and V comprising the ECM and interstitial matrix. The tumor microenvironment. Which is characterized by ECM deposition, remodeling, and cross-linking is crucial for PC progression, especially for tumor invasion (Piersma et al., 2020). Elevated levels of ECM proteins derived from tumor cells tend to correlate with poor survival of patients with PC (Tian et al., 2019). Hypoxia is also closely related to interstitial fibrosis. The deposition of ECM in PC can aggravate hypoxia and lead to HIF-1α upregulation. Hypoxia and HIF-1α can also increase the collagen levels and enhance the invasiveness of PC (Spivak-Kroizman et al., 2013; Li et al., 2016; Katagiri et al., 2018). A literature review showed that HIF-1α can upregulate the expression of TGFβ and activate the TGFβ/Smad pathway, resulting in the deposition of collagen in dermal fibrocytes, while Smad4 can promote the expression of HIF-1α (Mingyuan et al., 2017). HIF-1α can facilitate keloid progression by activating TGFβ (Lei et al., 2019). Collagen deposition in the heart and oral mucosa is also mediated by the HIF-1α/TGFβ pathway (Feng et al., 2021; Zhang et al., 2021). Other studies have found that the TGFβ/Smad3 pathway can increase the expression of HIF-1α and promote collagen deposition in renal epithelial cells (Basu et al., 2011; Rozen-Zvi et al., 2013). TGFβ1 can stabilize HIF-1α by producing reactive oxygen species and reducing hydroxylation, leading to alveolar epithelial-mesenchymal transformation (Zhou et al., 2009). HIF-1α and TGFβ have a positive feedback mechanism in promoting epithelial-mesenchymal transformation in hepatocellular carcinoma (Su et al., 2019).

Regarding the relationship between TQ and HIF-1α, TQ can promote the apoptosis of renal cancer cells through the HIF-1α-mediated glycolysis pathway, and its mechanism may be related to the inhibition of ubiquitination and heat shock protein 90 to reduce HIF-1α stability (Lee et al., 2019). The interaction between TQ and HIF-1α in PC has not been studied. In view of the anticancer, antifibrotic, and antioxidative effects of TQ, we speculate that TQ effects may be mediated by HIF-1α to regulate the TGFβ/Smad pathway and affect collagen levels in PC, thereby improving GEM sensitivity of PC. In this study, we examined this hypothesis *in vivo* and *in vitro* and analyzed possible underlying mechanisms.

## 2 Patients, materials, and methods

### 2.1 Patients

We collected samples from 30 patients with PC between January 2020 and December 2022 in the Qinghai Provincial People’s Hospital (The ethical clearance numbers for human inheritance study was 2021–106). The study population comprised 15 men and 15 women, aged 36–77 years, with TNM stages I, II, III, and IV in 6, 11, 2, and 11 patients, respectively. Inclusion criteria were a diagnosis of PC and complete tumor resection. Exclusion criteria were 1) patients with preoperative radiotherapy and chemotherapy, 2) patients with long-term use of hormone or immunosuppressive drugs before surgery, and 3) postoperative pathological confirmation of a non-pancreatic cancer.

### 2.2 Materials

TQ (HY-D0803, MCE); GEM (HY-17026, MCE); dimethyl sulfoxide (Sigma); fetal bovine serum (GIBCO), RPMI-1640 medium (GIBCO); primary antibodies including anti-COL1A1 (67288-1-Ig, PROTEINTECH), anti-COL3A1 (22734-1-AP, PROTEINTECH), anti-COL5A1 (67604-1-Ig, PROTEINTECH), anti-HIF-1α (20960-1-AP, PROTEINTECH), anti-OH-HIF-1α (3434T, CST), anti-TGFβ1 (21898-1-AP, PROTEINTECH), anti-Smad2 (12570-1-AP, PROTEINTECH), anti-Smad3 (66516-1-Ig, PROTEINTECH); hTGFβ (HY-P70543, MCE); LY2157299 (HY-13226, MCE); PX-478 (HY-10231, MCE); 26S proteasome inhibitor (MG132) (HY-132598, MCE); CCK-8 kit (Biyuntian Institute of Biotechnology); TUNEL Kit (11684817910, Roche); Annexin V-FITC Apoptosis Detection Kit (AO 2001-02P-G, Tianjin Sanjian Biotechnology Co., Ltd.) were used in this study. Phosphate-buffered saline (PBS) and tris buffered saline (TBS) were purchased from Gino Biomedical Technology Co. Ltd.

### 2.3 Immunohistochemistry

To dewax the paraffin slices, they were immersed in xylene for 15 min (two times), absolute ethanol for 5 min (two times), 90% alcohol for 5 min, 75% alcohol for 5 min, then washed with tap water. After washing the slices with pure water, they were placed into 0.01 M citrate buffer (pH 6.0), heated in the microwave for antigen repair, and repaired with medium high fire for 2–8 min. The time was adjusted for each sample. The samples were cooled to room temperature, and a circle was drawn around the tissue with a histochemical pen to prevent subsequent loss of incubation solutions. The samples were washed with PBS three times for 5 min each, then incubated with 3% H_2_O_2_ at room temperature for 15–30 min to block endogenous peroxidases, followed by another three PBS washes lasting 5 min each. The primary antibody was diluted in 5% BSA to the intended concentration, then an appropriate amount of the working solution containing the primary antibody was added, and the sample was kept at 4°C overnight. After reheating, the sample was washed with PBS three times for 5 min each. The working solution containing the secondary antibody was added, and the sample was incubated in a 37°C water bath for 30 min, followed by three PBS washing steps for 5 min each. After adding the DAB working solution, the development was controlled under the microscope until the color was brown-yellow, and the color development was stopped by washing the samples with tap water. Subsequently, samples were stained with hematoxylin for 3–10 min, differentiated with 1% hydrochloric acid, washed with tap water, 1% ammonia in water returning to blue, followed by another washing step with tap water. If the staining effect was insufficient, samples were treated with celestite blue dye solution for 3–5 min, and then stained with hematoxylin after tap water washing. Dehydration sealing film, dehydrate transparent, neutral gum sealing film.

### 2.4 Cell culture

The human PC cell line PANC-1 (20180418-01, BIOWING) were cultured in PMIS-1640 medium supplemented with 10% fetal bovine serum, 100 g/mL penicillin, and 100 g/mL streptomycin in an incubator (37°C, 5% CO_2_, saturated humidity). Adherent cell monolayers were grown to 70%–80% confluence and subcultured by trypsin digestion.

### 2.5 Cell viability assay

PANC-1 cells in the logarithmic growth phase were dissociated into a single-cell suspension, and 4×10^3^ cells per well were seeded into a 96-well plate. After cell attachment, the TQ and GEM intervention group and the untreated control group were established for subsequent experiments. The TQ intervention group was treated with different concentrations of TQ (5,10, 15, 20, 25, 30, and 35 μM) and GEM intervention group was treated with different concentrations of GEM (0.01, 0.1, 1, 10, 25, and 50 μM) and both incubated for 72 h, whereas the untreated control group received the same volume of PBS. At 1 h before the end of the drug treatment, CCK-8 was added to each well, and the cells were cultured for an additional 1 h. A microplate reader (BD AriaIII) was used to determine the absorption at the wavelength of 450 nm, and the inhibition was calculated as:

Inhibition rate% = [(control group blank) − (TQ group blank)]/(control group blank) × 100%

The experiment was repeated three times.

### 2.6 Western blots

The cells were washed three times with PBS precooled to 4°C, protein lysate was added, and the cells were lysed on ice for 30 min after shaking. Afterward, the cells were collected using a cell scraper and centrifuged at 4°C (12,000 rpm, 10 min), the supernatant was collected, and the protein concentration was determined using the BCA method. Subsequently, 50 μg of protein per lane was electrophoresed on sodium dodecyl sulfate-polyacrylamide gel (SDS-PAGE), then transferred onto membranes and blocked. Afterward, primary antibodies were added to the membrane and incubated at 4°C overnight. The membranes were washed with TBST three times, then the secondary antibody was added and incubated at room temperature for 2 h. The samples were again washed with TBST three times, then the electrochemiluminescence solution was added. The relative expression level of the target protein was expressed as the ratio of the gray value of the target protein band to the gray value of the β-actin band used as an internal reference. The expression level of the control group was set to 100%, and the relative expression level of each experimental group was calculated.

### 2.7 Transwell assay

Normal control (NC), TQ-treated, or GEM-treated cells in the logarithmic phase were brought into a suspension of 10^5^ cells/mL, 1 mL cell suspension was centrifuged at 1,500 rpm for 5 min, and the supernatant was discarded. After adding 1 mL of serum-free medium, 200 μL of well-mixed cell suspension was transferred into the transwell chamber. Then, 500 μL complete medium containing 10% FBS was added to the plate before transferring the chamber into the plate which was kept in an incubator at 37°C and 5% CO_2_ for 8 h (adjusted according to the experiment). To prepare the dye solution, 0.5% crystal violet solution was diluted with PBS 1:4 prior to use to create a 0.1% crystal violet dye solution. To stain the migrated cells, the chamber was removed, the culture medium was rinsed off with PBS, and the cells in the upper chamber were wiped off with a cotton swab. Then, the cells in the lower chamber were fixed with paraformaldehyde for 20 min, washed twice with PBS, and dyed with crystal violet for 10 min. The crystal violet dye was washed off the surface, and images of the migrated cells were taken under an inverted microscope. The matrix and culture medium were diluted in a 1:3 ratio, 50 μL was transferred into the transwell chamber, and the chamber was dried in the incubator for the invasion assay.

### 2.8 Apoptosis

Flow cytometry was performed using Annexin V-FITC/PI double staining. A total of 4×10^5^ PANC-1 cells in the logarithmic growth stage were seeded into 6-well culture plates. When the cells had grown to 90% confluence, TQ or GEM was added, and the cells were cultured for another 24 h. The cells were digested by trypsin, centrifuged at 1,000 rpm for 5 min, and washed twice with cold PBS. Cells were incubated at room temperature (20–25°C) for 15 min while avoiding light exposure. The cells were measured in a plate reader at an excitation wavelength of 488 nm and an emission wavelength of 530 nm.

### 2.9 Construction of the plasmid vectors

HIF-1α cDNA cloning vector (with cleavage site) was purchased (ELK Biotechnology), and EcoR I and BamH I were used to cleave the cloning vectors. The cleavage products were recovered with a kit and detected by electrophoresis. The culture medium was centrifuged to collect the bacteria and remove the supernatant. Successively, 250 µL of Solution A, Solution B, and Solution C were added, then the sample was centrifuged to remove the supernatant. After adding 1/10 volume of the Endo-Remove Buffer solution, the sample was centrifuged, and the supernatant was removed. Anhydrous ethanol three times the sample volume was added, mixed with the sample, transferred to a P adsorption column, centrifuged, and the waste liquid was discarded. A total of 600 µL wash buffer solution was added and centrifuged, the wash buffer residue was discarded, and the remaining ethanol was removed. After adding 50 µL of elution buffer solution to the adsorption column, the sample was centrifuged, and the plasmid was extracted. The recombinant overexpressed vector was constructed by culturing the linked products. The cultured bacterial solution was analyzed by PCR, and 20 µL plasmid DNA was sequenced.

### 2.10 Terminal deoxynucleotidyl transferase dUTP nick end labeling (TUNEL) staining

Dewaxing paraffin sections to water: sections were immersed in 1) xylene 15 min; 2) xylene 15 min; 3) anhydrous ethanol 5 min; 4) anhydrous ethanol 5 min; 5) 90% alcohol; and 6) 75% alcohol 5 min then washed with tap water. Sections were then washed with pure water and circles drawn around the tissue with a tissue pen to prevent the incubation liquid from escaping in the following process. Sections were washed three times in PBS for 5 min each time. Slides were incubated with a prepared working solution of protease K in a 37°C water bath for 30 min, then washed in PBS three times for 5 min each time. Slides were then incubated with a prepared membrane breaking solution at room temperature for 10 min, then washed three times with PBS for 5 min each time. Reagents 1 and 2 in the TUNEL kit were mixed at 1:10 to prepare an appropriate incubation solution and slides were incubated with the solution at 37°C for 1 h–1.5 h or at 4°C overnight. Sections were washed with PBS three times for 5 min each time, DAPI nucleation drops were added, and samples were incubated at room temperature (20–25°C) and in the dark for 20–30 min then washed with PBS. The tablets were sealed with anti-fluorescence quenching tablets, observed and photographed under a microscope.

### 2.11 *In vivo* experiments

PANC-1 cells cultured under normoxic conditions formed subcutaneous tumors in nude mice, with a tumorigenic cycle of 4 weeks (grouping: a total of 9 mice were divided into three groups with 3 mice per group; age: 6– weeks; weight: 200–220 g). The ethical clearance numbers and other details for animal study was ZX-63000001-2023-053. NC mice were treated with 0.2 mL 1% ethanol by oral gavage. GEM was intraperitoneally injected (100 mg/kg) twice a week for 2 weeks. TQ was administered by oral gavage (20 mg/kg) once a day for 2 weeks. The nude mice were killed by cervical dislocation 8 weeks later, the tumor was removed, and tumor volume and weight were measured.

### 2.12 Statistical analysis

All data obtained in this study were statistically analyzed using SPSS 26.0 software, and measurement data were expressed as mean ± standard deviation. The *t*-test was performed, and *p* < 0.05 was considered to indicate a statistical difference between groups.

## 3 Results

### 3.1 HIF-1α, TGFβ1, COL1A1, COL3A1, and COL5A1 expression in cancerous and para-carcinoma tissues of PC

**FIGURE 1:**
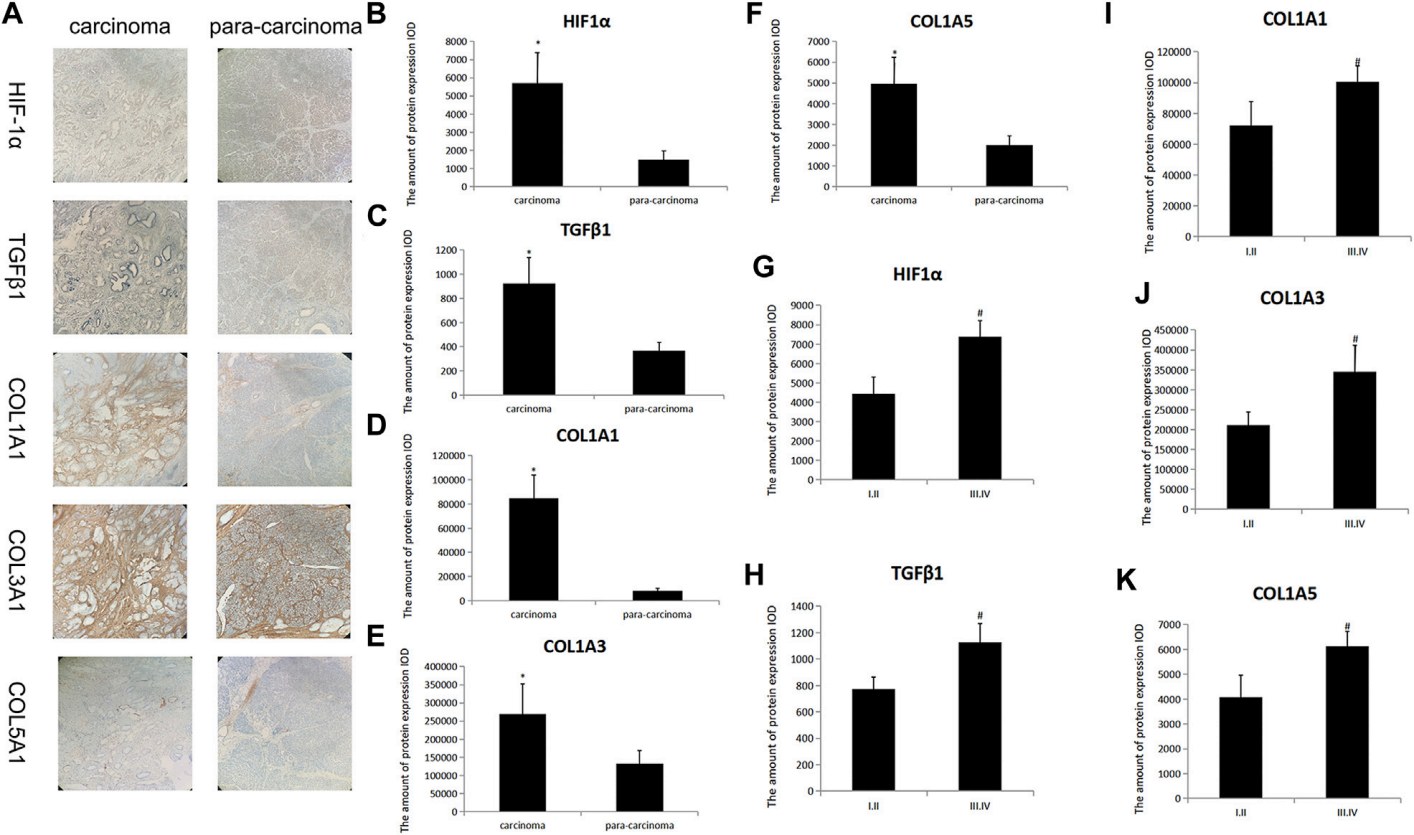
HIF-1α, TGFβ1, COL1A1, COL3A1, and COL5A1 expression in carcinoma and para-carcinoma tissues of patients with PC. **(A)** HIF-1α, TGFβ1, COL1A1, COL3A1, and COL5A1 pathological images of carcinoma and para-carcinoma tissues in PC. **(B–F)** Statistical analysis of the HIF-1α **(B)**, TGFβ1 **(C)**, COL1A1 **(D)**, COL3A1 **(E)**, and COL5A1**(F)** expression in carcinoma and para-carcinoma tissues of PC. **(G–K)** Statistical analysis of the HIF-1α **(G)**, TGFβ1 **(H)**, COL1A1 **(I)**, COL3A1 **(J)**, and COL5A1 **(K)** expression at different TNM stages in PC. *p < 0.05 compared with para-carcinoma tissues; #*p* < 0.05 compared with stages I/II (*n* = 3).

Immunohistochemistry was used to compare the expression levels of HIF-1α, TGFβ1, COL1A1, COL3A1, and COL5A1 in PC and its surrounding tissues. The expression levels of these proteins in the cancer tissues were significantly higher than those in para-cancer tissues (*t*-test, all *p* < 0.05; Figures 1A–F). Moreover, these expression levels were closely related to the TNM stage of the patient (*t*-test, all *p* < 0.05; Figures 1G–K). These results suggest that HIF-1α, TGFβ1, COL1A1, COL3A1, and COL5A1 may be related to the occurrence and development of PC and that they can provide a reference for the degree of PC malignancy.

### 3.2 TQ and GEM concentration-dependently influence the viability of PC cells

**FIGURE 2:**
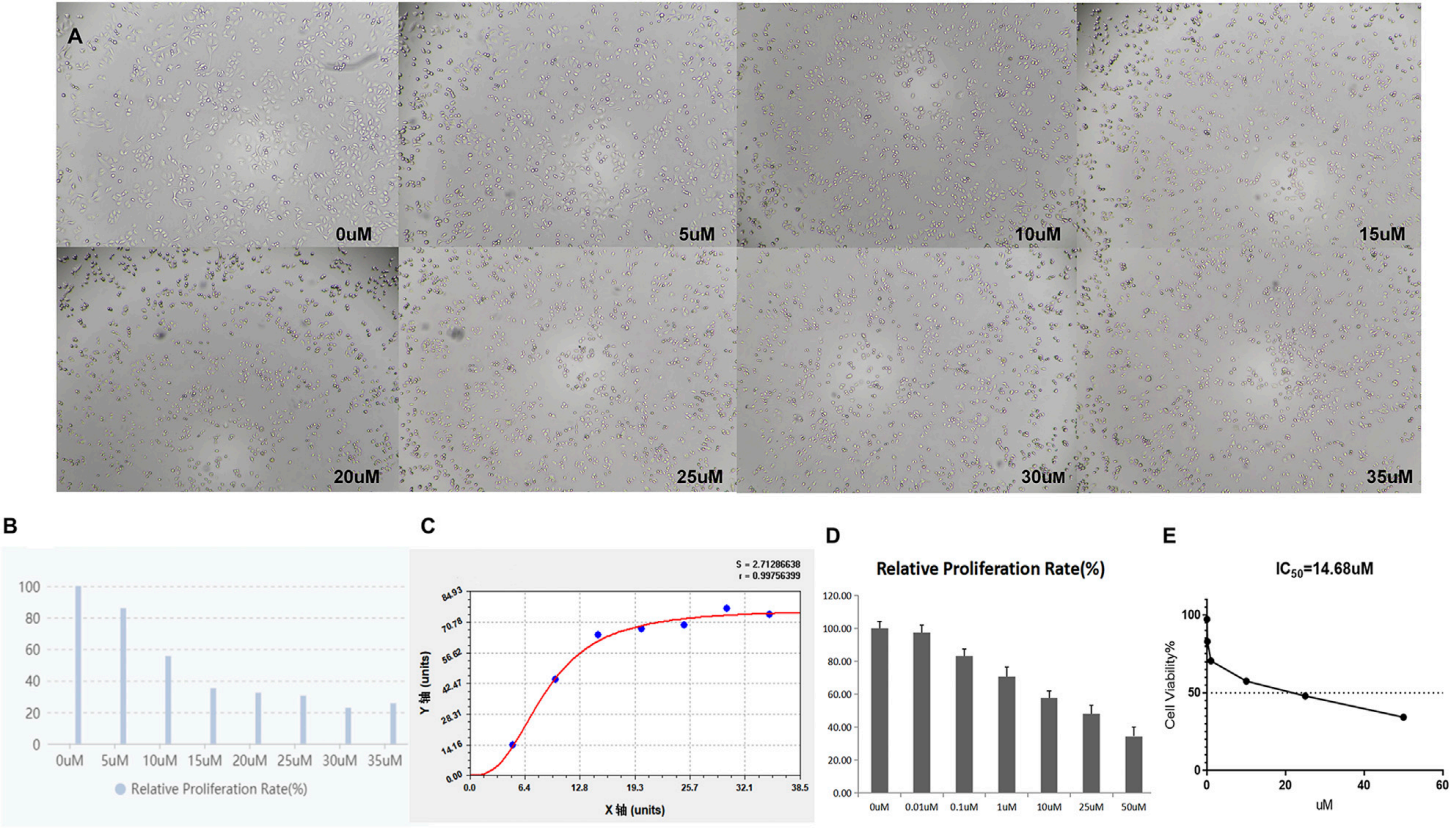
TQ at various concentrations influences the viability of PC cells under hypoxic conditions. **(A)** Morphology of TQ-treated PC cells in the CCK-8 assay. **(B)** Relative proliferation rate of TQ-treated PC cells in the CCK-8 assay. **(C)** Proliferation curve of TQ-treated PC cells in the CCK-8 assay. **(D)** Relative proliferation rate of GEM-treated PC cells in the CCK-8 assay. **(E)** Cell viability of GEM-treated PC cells in the CCK-8 assay (*n* = 3).

First, we examined whether TQ and GEM can influence the proliferation of PC cells. As HIF-1α is only detectable under hypoxic conditions, these experiments were performed in 1% hypoxic conditions with a treatment time of 8 h. As shown in Figures 2A–C, CCK-8 assays were applied to test the potential effects of TQ on the viability of PANC-1 cells. Increasing TQ concentrations showed a progressive reduction in the number of PANC-1 cells compared to the number of untreated cells suggesting that TQ impaired the proliferation of PC cells under different concentrations. As 11 μM was the half-maximal inhibitory concentration (IC_50_) of this compound, we applied this concentration in subsequent experiments. In addition, CCK-8 assays were applied to test GEM effects on the viability of PANC-1 cells (Figures 2D,E). GEM administration resulted in a concentration-dependent inhibition of PANC-1 cell proliferation. We considered 14.68 µM as the GEM concentration in subsequent experiments, as this was the IC_50_ value of this compound.

### 3.3 HIF-1α expression in TQ-treated PC cells under normoxic and hypoxic conditions

**FIGURE 3:**
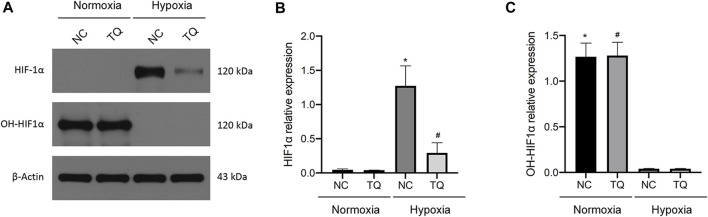
Effect of TQ on HIF-1α expression under normoxic and hypoxic conditions. **(A)** HIF-1α and OH-HIF-1α expression of normal control (NC) or TQ-treated PC cells in normoxia or hypoxia. **(B)** Statistical analysis of the HIF-1α expression in NC or TQ-treated PC cells under normoxic and hypoxic conditions. *p < 0.05 compared with NC in normoxia; #*p* < 0.05 compared with NC in hypoxia. **(C)** Statistical analysis of OH-HIF-1α expression in NC or TQ-treated PC cells under normoxic or hypoxic conditions. *p < 0.05 compared with NC in hypoxia; #*p* < 0.05 compared with TQ in hypoxia (*n* = 3).

Next, we investigated the relationship between TQ administration and HIF-1α expression. The treatment time was 8 h. In the presence and absence of TQ, PANC-1 cells exhibited different expression patterns under normoxic and hypoxic conditions. Almost no expression of HIF-1α was observed in PANC-1 cells under normoxic conditions, whereas HIF-1α expression was increased in NC PANC-1 cells and decreased in TQ-treated PANC-1 cells under hypoxic conditions (*t*-test, *p* < 0.05). The hydroxylated HIF-1α form OH-HIF-1α was significantly more expressed under normoxic conditions compared to hypoxic (*t*-test, *p* < 0.05), and its expression remained stable regardless of TQ administration (Figures 3A–C). These results suggest a potential role of TQ in regulating HIF-1α in PC under hypoxic conditions, but not in normoxic conditions.

### 3.4 TQ regulates ECM production through the TGFβ/Smad pathway in PC cells under hypoxic conditions

**FIGURE 4:**
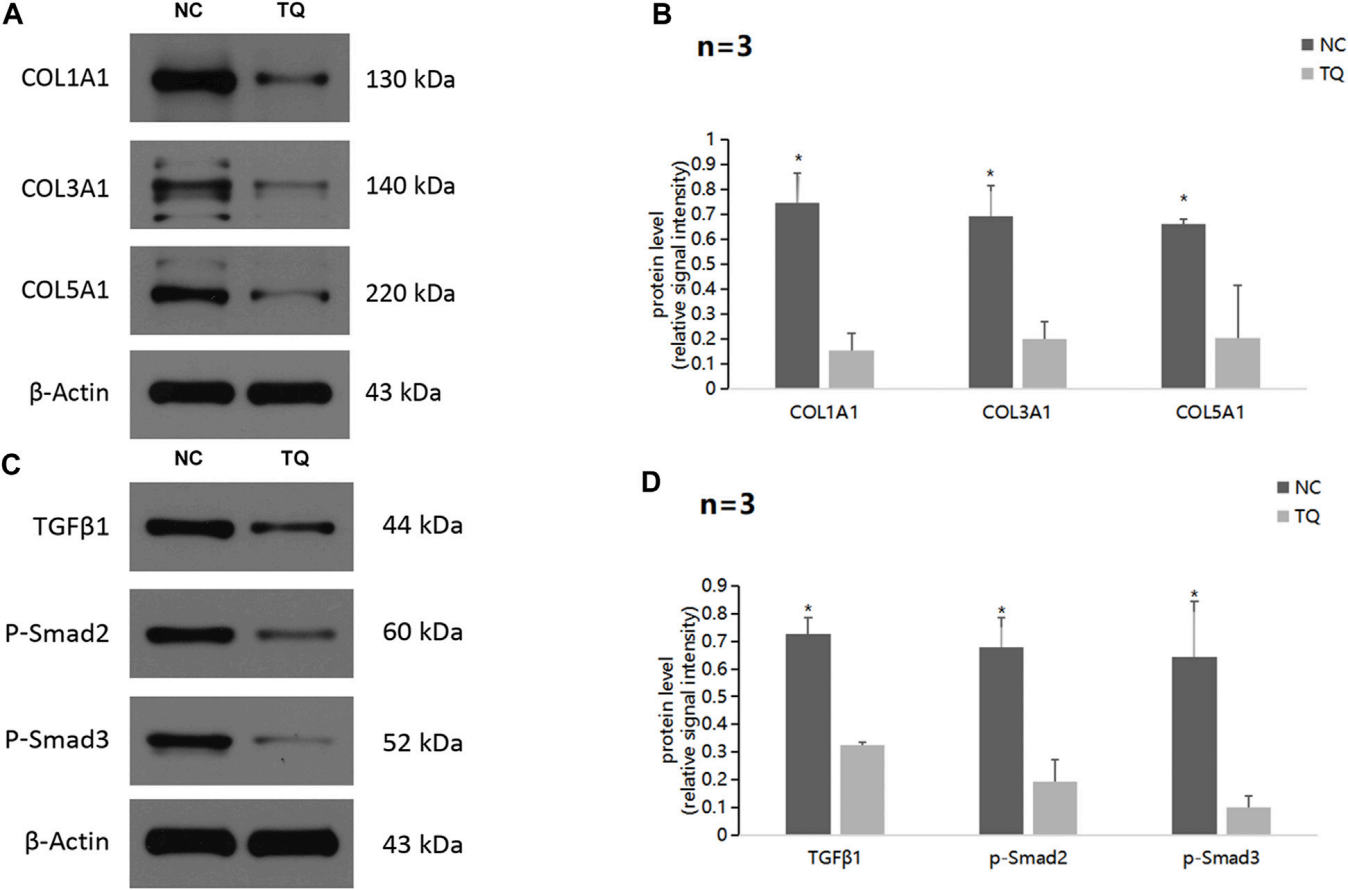
TQ influences ECM production through the TGFβ/Smad pathway in PC cells under hypoxic conditions. **(A)** Western blot of ECM production-related proteins in NC or TQ-treated PANC-1 cells under hypoxic conditions. **(B)** Statistical analysis of **(A)**. **(C)** Western blot of TGFβ pathway-related proteins in NC or TQ-treated PANC-1 cells under hypoxic conditions. **(D)** Statistical analysis of **(C)**. *p < 0.05 compared with TQ (*n* = 3).

Next, we focused on the potential mechanism by which TQ influences the functions of PC cells. Western blot assays showed a significant decrease in the ECM production pathway-related proteins COL1A1, COL3A1, and COL5A1 under hypoxic conditions in TQ-treated PANC-1 cells (The treatment time was 8 h) (*t*-test, *p* < 0.05; Figures 4A,B). These results suggest a regulatory role of TQ in remodeling the ECM of the tumor microenvironment, which may influence the progression of PC. Moreover, we found that TQ can also decrease the expression of TGFβ1, p-Smad2 and p-Smad3 which are key proteins of the TGFβ/Smad signaling pathway in PANC-1 cells (*t*-test, *p* < 0.05; Figures 4C,D).

**FIGURE 5:**
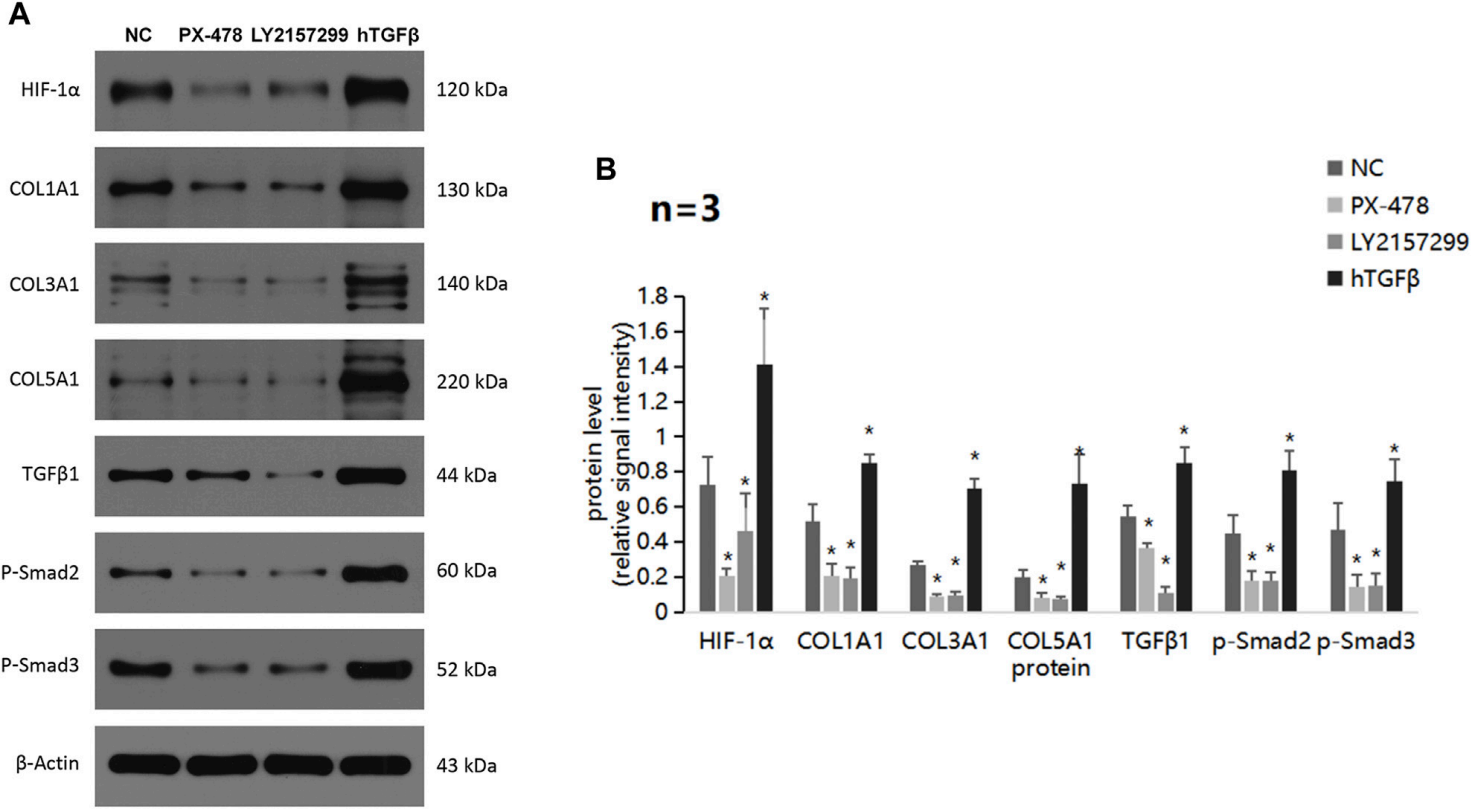
ECM production and TGFβ pathway alterations by a HIF-1α inhibitor, TGFβ1 receptor inhibitor, and recombinant hTGFβ in PC cells under hypoxic conditions. **(A)** Western blot of ECM production-related proteins and TGFβ pathway-related proteins in PC cells treated with a HIF-1α inhibitor, TGFβ1 receptor inhibitor, or recombinant hTGFβ under hypoxic conditions. **(B)** Statistical analysis of **(A)**. *p < 0.05 compared with NC (*n* = 3).

Furthermore, we evaluated the level of ECM production pathway-related proteins and TGFβ/Smad signaling pathway-related proteins in PANC-1 cells treated with a HIF-1α inhibitor, TGFβ1 receptor inhibitor, or recombinant hTGFβ (Figures 5A,B). The experimental condition was normoxic and the treatment time was 24 h. Strikingly, all proteins showed significant decreases in expression when treated with the HIF-1α inhibitor PX-478 and the TGFβ1 receptor inhibitor LY2157299, but a remarkable upregulation when treated with recombinant hTGFβ. These results suggest that TQ may regulate the TGFβ/Smad pathway via HIF-1α, thereby affecting ECM production. In addition, HIF-1α and TGFβ1 have positive feedback loops in regulating ECM production.

### 3.5 TQ and GEM affect cell apoptosis and motility of PC cells

**FIGURE 6:**
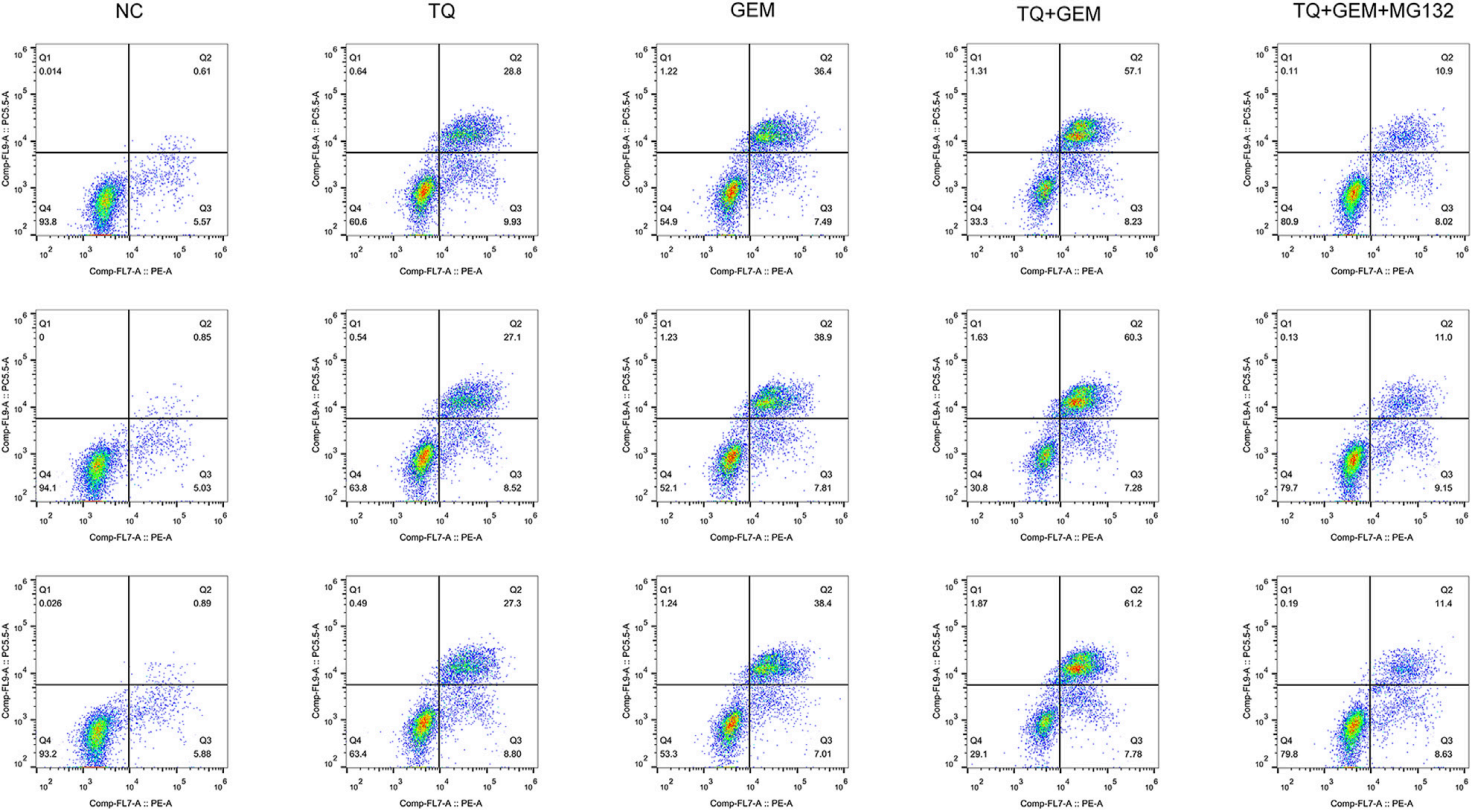
Analysis of apoptosis in NC, TQ-, GEM-, TQ + GEM-, and TQ + GEM + MG132-treated PANC-1 cells under hypoxic conditions (*n* = 3).

**FIGURE 7:**
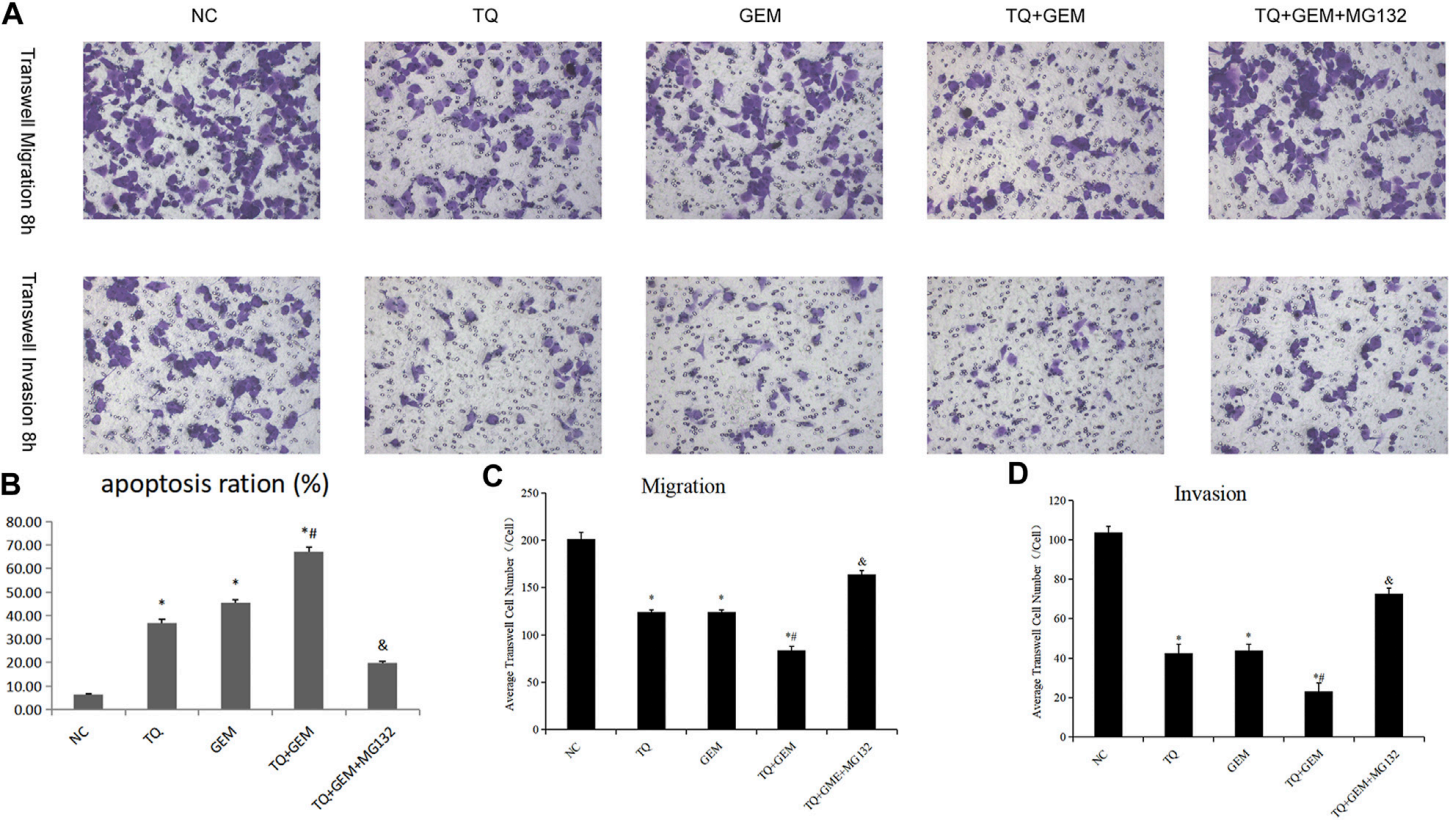
TQ and GEM affect cell apoptosis and motility in PC cells under hypoxic conditions. **(A)** Transwell (migration and invasion) of NC, TQ-, GEM-, TQ + GEM-, and TQ + GEM + MG132-treated PANC-1 cells under hypoxic conditions. **(B)** Statistical analysis of Figure 6 (apoptosis analysis). **(C)** Statistical analysis of **(A)** (transwell migration). **(D)** Statistical analysis of **(A)** (transwell invasion). *p < 0.05 compared with NC; #*p* < 0.05 compared with TQ or GEM; and *p* < 0.05 compared with TQ + GEM (*n* = 3).

Afterward, we examined whether TQ and GEM affect cell apoptosis processes and motility during PC progression. We observed after 8 h of hypoxia (1% O_2_) the results in five experimental groups, namely, the NC, TQ, GEM, TQ + GEM, and TQ + GEM + MG132 (a reagent that blocks the effect of TQ on HIF-1α, 20 µM) groups (Lee et al., 2019). Based on flow cytometry and transwell assays, we found that both TQ and GEM significantly promoted apoptosis and inhibited the invasion and migration of PANC-1 cells (*t*-test, all *p* < 0.05). Moreover, the combination of TQ and GEM was more effective than either TQ or GEM alone (*t*-test, all *p* < 0.05). In the presence of MG132, the effects of the combination TQ + GEM on promoting apoptosis and inhibiting invasion and migration were significantly decreased (*t*-test, *p* < 0.05; Figures 6, 7). This suggests that the synergistic effect of TQ and GEM may be mediated by HIF-1α.

### 3.6 TQ might affect GEM sensitivity by regulating ECM production in PC cells under hypoxic conditions

**FIGURE 8:**
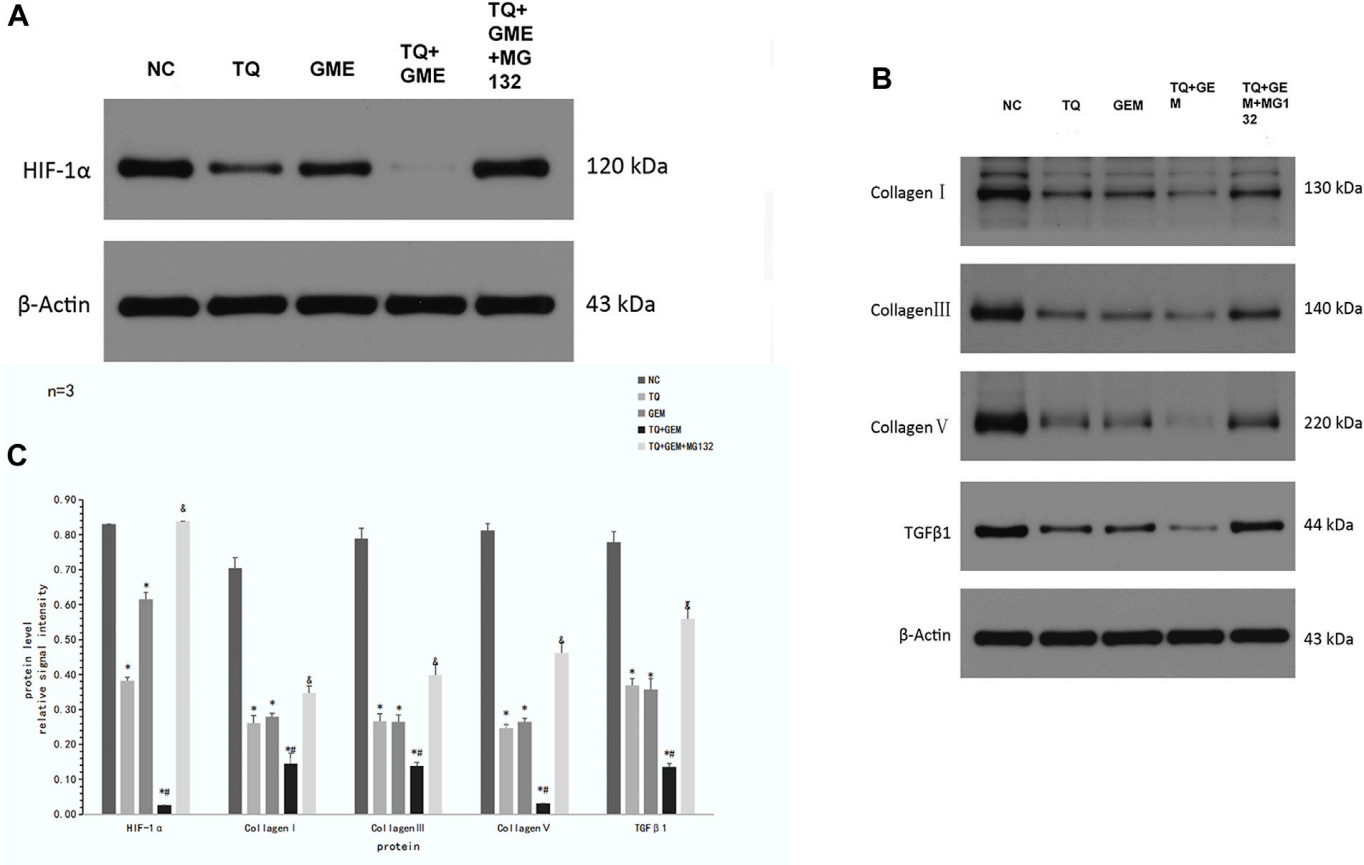
TQ might affect GEM sensitivity by regulating ECM production in PC cells under hypoxic conditions. **(A)** Western blot of HIF-1α in NC, TQ-, GEM-, TQ + GEM-, and TQ + GEM + MG132-treated PANC-1 cells under hypoxic conditions. **(B)** Western blot of ECM production-related proteins and TGFβ1 in NC, TQ-, GEM-, TQ + GEM-, and TQ + GEM + MG132-treated PANC-1 cells under hypoxic conditions. **(C)** Statistical analysis of **(A**, **B)**. *p < 0.05 compared with NC; #*p* < 0.05 compared with TQ or GEM; and *p* < 0.05 compared with TQ + GEM (*n* = 3).

As we demonstrated that TQ can enhance the toxic effect of GEM and preliminarily found that TQ can regulate ECM production in PC cancer, we further investigated the relationship between the TQ effect on GEM sensitivity and ECM in PC. Using the same experimental groups and conditions as mentioned above, Western blot assays were used to compare changes in HIF-1α levels. HIF-1α was significantly downregulated in the TQ group compared to the NC group (*t*-test, *p* < 0.05). In addition, HIF-1α was significantly downregulated in the TQ + GEM group compared to the GEM group (*t*-test, *p* < 0.05). Moreover, the HIF-1α level was significantly upregulated in the TQ + GEM + MG132 group compared to the TQ + GEM group (*t*-test, *p* < 0.05; Figures 8A,C).

To determine the relationship between TQ effects on GEM sensitivity and collagen expression, COL1A1, COL3A1, COL5A1, and TGFβ1 levels were determined by Western blot assays. These proteins showed the same expression trends as HIF-1α in all experimental groups (*t*-test, *p* < 0.05; Figures 8B,C). These results suggest that TQ may affect GEM sensitivity by influencing ECM production, and HIF-1α may play an important role in this process.

### 3.7 HIF-1α plays an important role in ECM production and affects apoptosis and migration of PC cells

**FIGURE 9:**
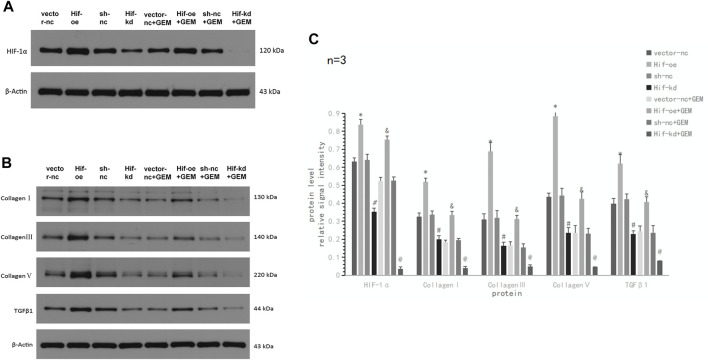
HIF-1α affects TGFβ1 and ECM production-related proteins in PC cells under hypoxic conditions. **(A)** Western blot of HIF-1α in PANC-1 cells treated with vector-nc, Hif-oe, sh-nc, Hif-kd, vector-nc + GEM, Hif-oe + GEM, sh-nc + GEM, or Hif-kd + GEM under hypoxic conditions. **(B)** Western blot of ECM production-related protein and TGFβ1 in PANC-1 cells treated with vector-nc, Hif-oe, sh-nc, Hif-kd, vector-nc + GEM, Hif-oe + GEM, sh-nc + GEM, or Hif-kd + GEM under hypoxic conditions. **(C)** Statistical analysis of **(A**, **B)**. *p < 0.05 compared with vector-nc; #*p* < 0.05 compared with sh-nc; and *p* < 0.05 compared with vector-nc + GEM; @ *p* < 0.05 compared with sh-nc + GEM (*n* = 3).

Although we demonstrated that TQ can inhibit HIF-1α expression and enhance GEM sensitivity and found that HIF-1α may play a key role in this process, we did not directly demonstrate the effect of HIF-1α on GEM sensitivity. Therefore, we performed HIF-1α over-expression and knockdown experiments in PANC-1 cells using the plasmid transfer method to compare the apoptosis and migratory abilities of PANC-1 cells. We observed the results after 8 h of hypoxia (1% O_2_) in eight experimental groups: vector-nc (vector normal control) group, Hif-oe (HIF-1α over expression) group, sh-nc (shRNA normal control) group, Hif-kd (HIF-1α knockdown) group, vector-nc + GEM group, Hif-oe + GEM group, sh-nc + GEM group, and Hif-kd + GEM group. Using Western blotting, we found that the HIF-1α levels in HIF-1α over-expression groups were significantly higher than those in the corresponding control groups (Hif-oe group > vector-nc group, *t*-test, *p* < 0.05; Hif-oe + GEM group > vector-nc + GEM group, *t*-test, *p* < 0.05), whereas the HIF-1α knockdown groups had significantly lower levels than the control groups (Hif-kd group < sh-nc group, *t*-test, *p* < 0.05; Hif-kd + GEM group < sh-nc + GEM group, *t*-test, *p* < 0.05; Figures 9A,C). We also measured the expression of COL1A1, COL3A1, COL5A1, and TGFβ1 in each group, and these proteins showed the same expression trend as HIF-1α in all groups (*t*-test, all *p* < 0.05; Figures 9B,C).

**FIGURE 10:**
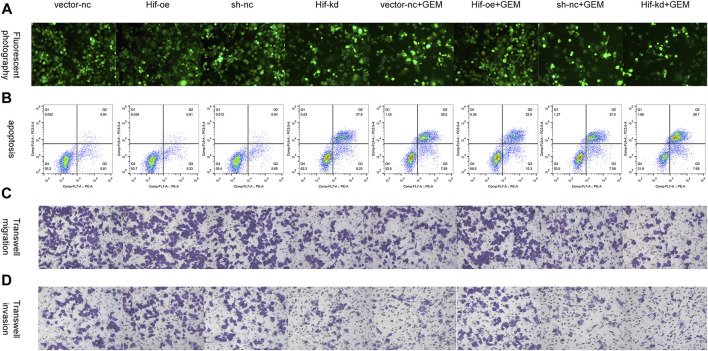
HIF-1α affects cell apoptosis and motility in PC cells under hypoxic conditions. **(A)** Tunel fluorescence staining marked apoptosis of PANC-1 cells treated with vector-nc, Hif-oe, sh-nc, Hif-kd, vector-nc + GEM, Hif-oe + GEM, sh-nc + GEM, or Hif-kd + GEM under hypoxic conditions. **(B)** Apoptosis of PANC-1 cells treated with vector-nc, Hif-oe, sh-nc, Hif-kd, vector-nc + GEM, Hif-oe + GEM, sh-nc + GEM, or Hif-kd + GEM under hypoxic conditions. **(C)** Transwell migration of PANC-1 cells treated with vector-nc, Hif-oe, sh-nc, Hif-kd, vector-nc + GEM, Hif-oe + GEM, sh-nc + GEM, or Hif-kd + GEM under hypoxic conditions. **(D)** Transwell invasion of PANC-1 cells treated with vector-nc, Hif-oe, sh-nc, Hif-kd, vector-nc + GEM, Hif-oe + GEM, sh-nc + GEM, or Hif-kd + GEM under hypoxic conditions (*n* = 3).

**FIGURE 11:**
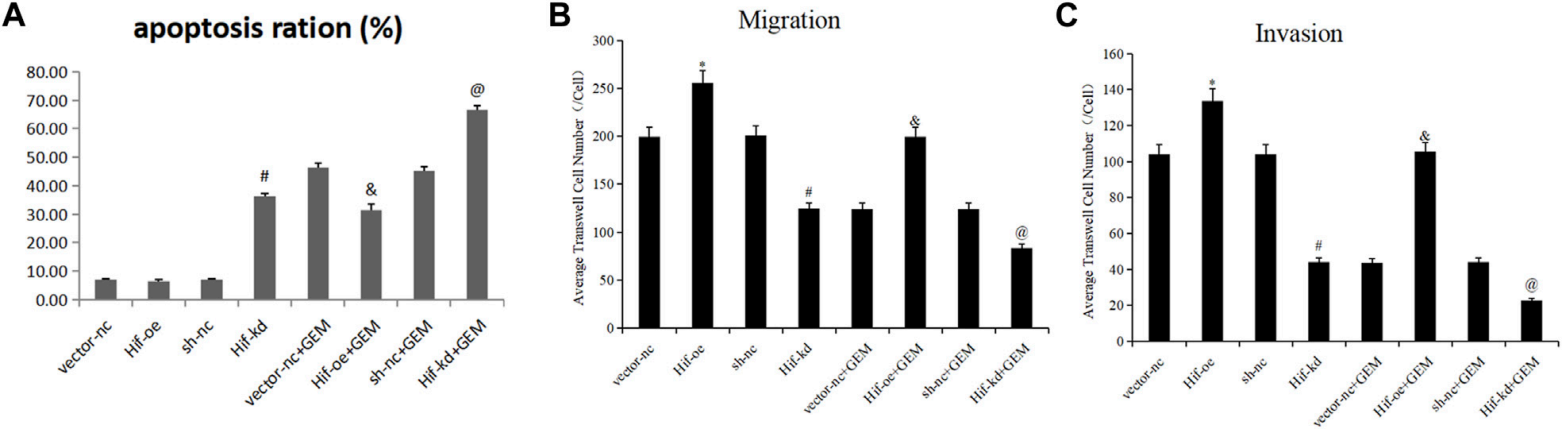
Statistical analysis of HIF-1α-affected cell apoptosis and motility in PC cells under hypoxic conditions. **(A)** Statistical analysis of Figure 10B. **(B)** Statistical analysis of Figure 10C. **(C)** Statistical analysis of Figure 10D. *p < 0.05 compared with vector-nc; #*p* < 0.05 compared with sh-nc; and *p* < 0.05 compared with vector-nc + GEM; @ *p* < 0.05 compared with sh-nc + GEM (*n* = 3).

To verify the effect of HIF-1α on apoptosis and motility of PC cells, we performed flow cytometry apoptosis and transwell assays. In the absence of GEM, HIF-1α over-expression had little effect on the apoptosis of PANC-1 cells (*t*-test, *p* > 0.05), but HIF-1α knockdown significantly promoted the apoptosis of PANC-1 cells (*t*-test, *p* < 0.05; Figures 10A,B; Figure 11A). In the presence of GEM, HIF-1α over-expression inhibited the apoptosis of PANC-1 cells (*t*-test, *p* < 0.05), whereas HIF-1α knockdown promoted apoptosis (*t*-test, *p* < 0.05; Figure 10B; Figure 11A). In the transwell assay, HIF-1α over-expression improved the motility of PANC-1 cells regardless of GEM presence (*t*-test, *p* < 0.05), whereas HIF-1α knockdown reduced the motility of PANC-1 cells (*t*-test, *p* < 0.05; Figures 10C,D; Figures 11B,C). These results suggest that HIF-1α plays a key role in enhancing GEM sensitivity and is closely related to ECM production.

### 3.8 TQ with GEM suppresses tumor growth *in vivo*

**FIGURE 12:**
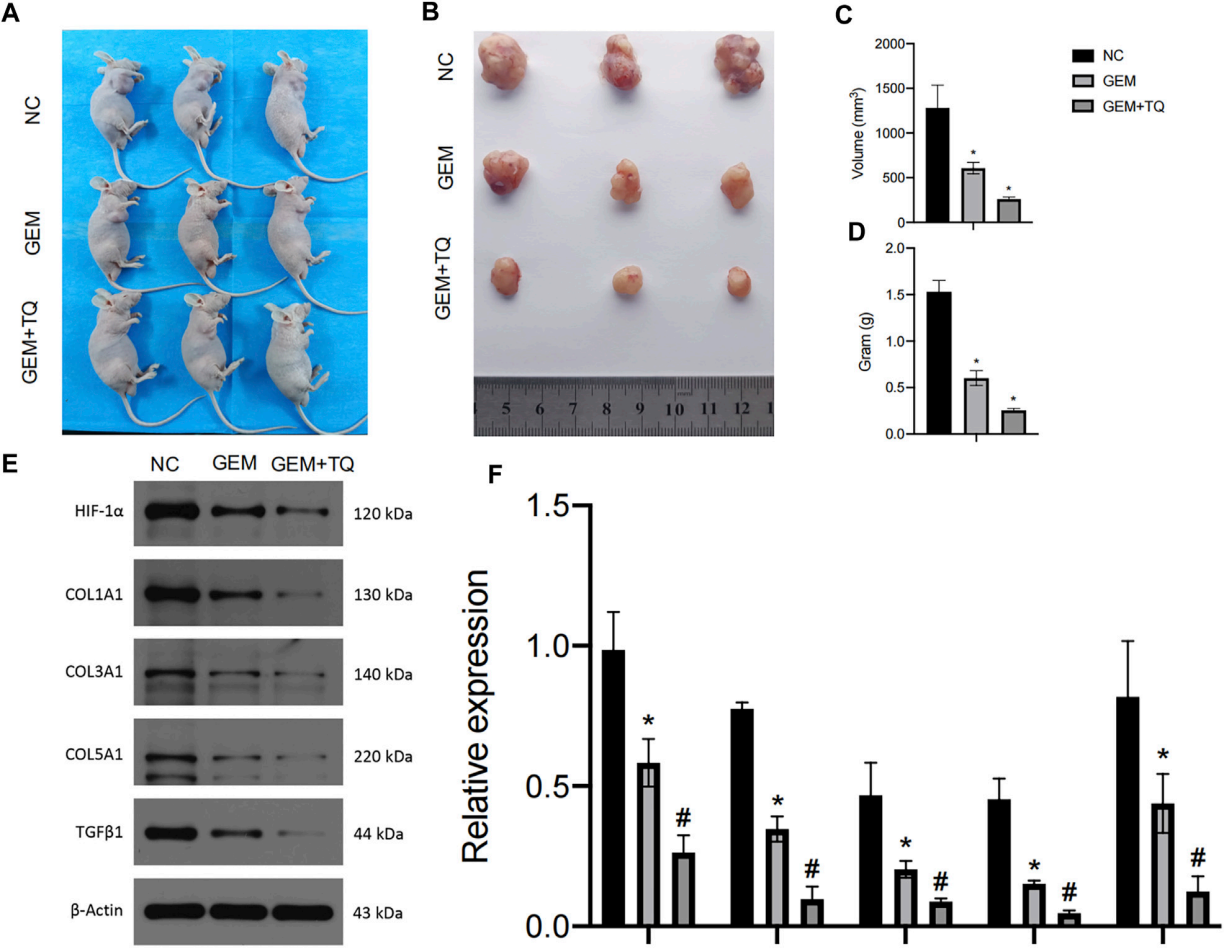
*In vivo* experiments reveal synergistic effects of GEM and TQ on PC cells. **(A)** General picture of NC, GEM-, and GEM + TQ-treated subcutaneous PC cell-bearing mice. **(B)** Subcutaneous tumors of NC, GEM-, and GEM + TQ-treated PC cell-bearing mice. **(C)** Statistical analysis of the tumor volume in **(B)**. **(D)** Statistical analysis of the tumor weight in **(B)**. **(E)** Western blot of ECM production and TGFβ pathway-related proteins in NC, GEM-, and GEM + TQ-treated subcutaneous PC cell tumors. **(F)** Statistical analysis of **(E)**. *p < 0.05 compared with NC; #*p* < 0.05 compared with NC or GEM (*n* = 3).

Finally, we examined whether TQ had corresponding *in vivo* antitumor effects in PC. PANC-1 cells were cultured under normoxic conditions for the generation of a subcutaneous tumorigenesis model in nude mice over a period of 28 days. GEM (100 mg/kg intraperitoneally twice a week for 2 weeks) and TQ (oral gavage 20 mg/kg once a day for 2 weeks) were used to treat tumor-bearing mice, whereas NC received 0.2 mL 1% ethanol by intragastric administration. The nude mice were killed by cervical dislocation 8 weeks later. Compared to the NC group, tumor volume and tumor weight were significantly lower in GEM-treated mice (*t*-test, both *p* < 0.05; Figures 12A–D). Furthermore, tumor volume and tumor weight were significantly lower when mice were treated with GEM + TQ compared to either NC or GEM alone (*t*-test, all *p* < 0.05; Figures 12A–D). This indicates a drug-enhancing effect of TQ interacting with GEM.

**FIGURE 13:**
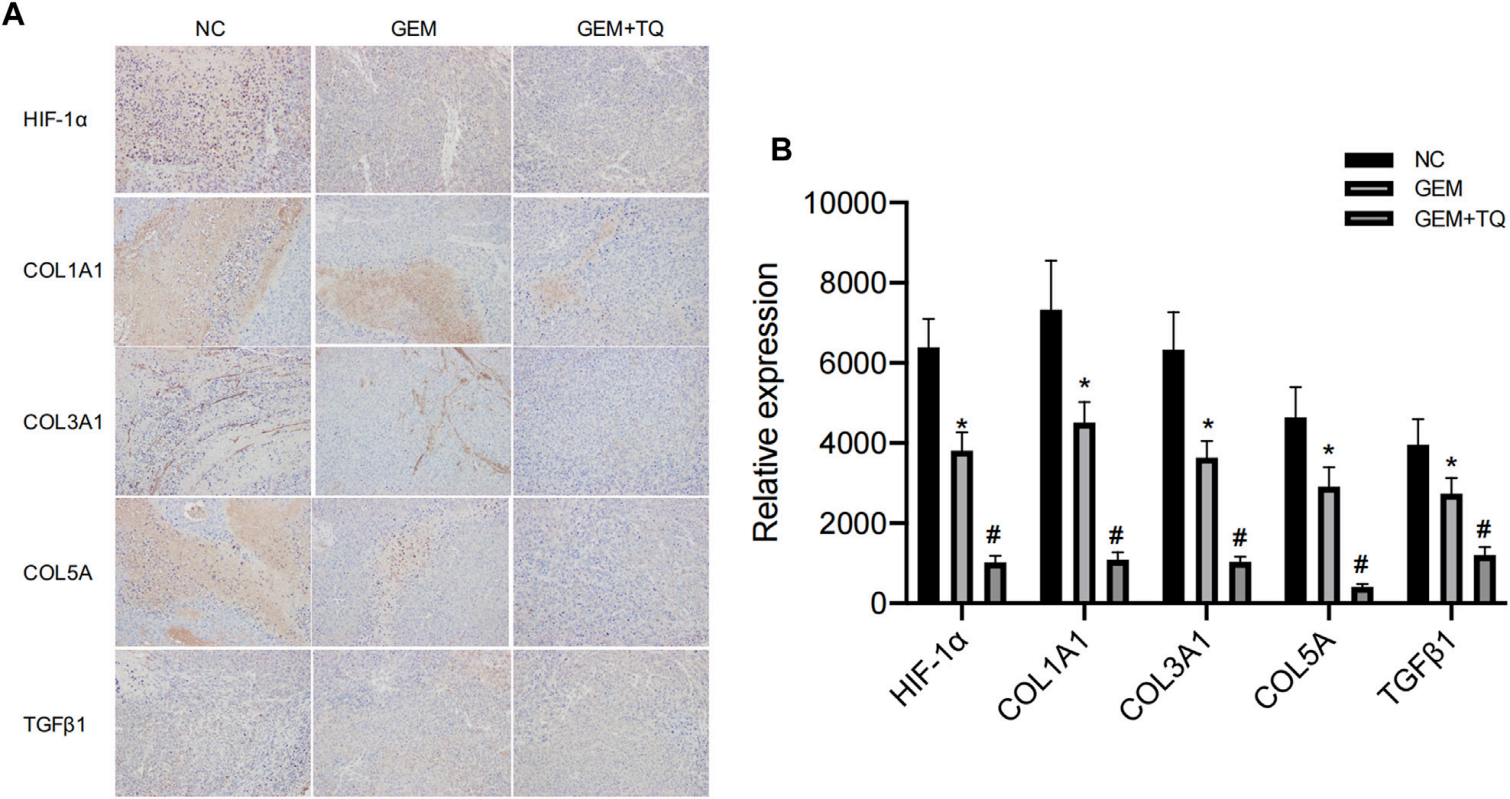
*In vivo* experiments reveal ECM production and TGFβ pathway alterations of GEM or TQ on PC cells. **(A)** Immunohistochemistry of ECM production and TGFβ pathway-related proteins in NC, GEM-, and GEM + TQ-treated subcutaneous PC cell tumors. **(B)** Statistical analysis of **(A)**. *p < 0.05 compared with NC; #*p* < 0.05 compared with NC or GEM (*n* = 3).

**FIGURE 14:**
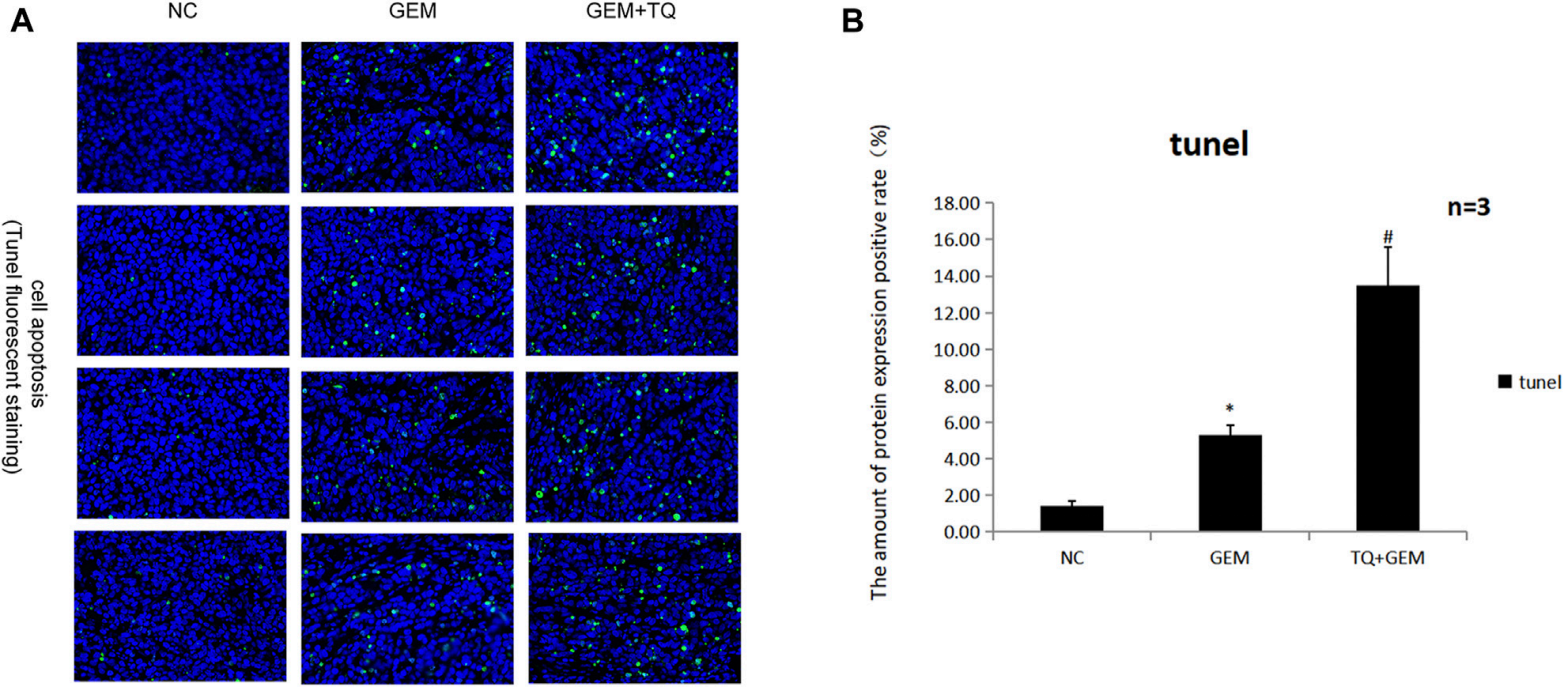
*In vivo* experiments reveal the effects of GEM and TQ on PC cell apoptosis. **(A)** Tunel fluorescence staining to detect the effects of TQ and GEM on apoptosis. **(B)** Statistical analysis of **(A)**. *p < 0.05 compared with NC; #*p* < 0.05 compared with NC or GEM (*n* = 3).

Similarly, we found that during GEM treatment, HIF-1α, ECM production pathway-related proteins, and TGFβ/Smad signaling pathway-related proteins were significantly downregulated (*t*-test, *p* < 0.05), as confirmed by Western blotting (Figures 12E,F) and immunohistochemistry (Figures 13A,B). Moreover, these protein levels were further decreased in the TQ + GEM group compared to the NC and GEM groups (*t*-test, *p* < 0.05). Furthermore, TUNEL fluorescence staining was used to detect the apoptosis of tumor cells in each group. Cell apoptosis was significantly higher in animals treated with GEM compared to NC animals, but even higher in the GEM + TQ group compared to the NC and GEM groups (*t*-test, all *p* < 0.05; Figures 14A,B). Together, these *in vivo* results verified the hypothesis that TQ influenced PC progression by regulating ECM production through the TGFβ/Smad pathway under hypoxic conditions.

## 4 Discussion

PC is one of the most malignant tumors owing to its high proliferation rate, high metastasis rate, and high recurrence rate (Li et al., 2019; Klein, 2021). Since most patients missed the opportunity for successful surgery at the time of diagnosis, clinical treatment mainly depends on pharmacological therapy. However, the 5 years survival time of patients with PC remains lower than 8% due to the lack of effective therapeutic drugs (Cai et al., 2021). Although various new strategies have been proposed, their therapeutic effects are not satisfactory (Neoptolemos et al., 2018; Torphy et al., 2020). At present, pharmacological treatment of PC mainly relies on chemotherapy, especially GEM-based regimens. Unfortunately, GEM-based chemotherapies have limited impact on PC due to their dose-limiting toxicity to normal tissues and increased chemoresistance. Drugs that can improve PC chemosensitivity to GEM are urgently needed.

Current research on TQ effects on PC has attracted attention, especially in improving the chemosensitivity of PC. Induction of apoptosis and loss of cell viability are the two main mechanisms by which traditional chemotherapeutic drugs kill cancer cells. To solve the problem of GEM resistance, Mu et al. found that pretreatment with TQ significantly enhanced apoptosis and growth inhibition by GEM in PC (Mu et al., 2014; Mu et al., 2015). Four possible mechanisms have been suggested. First, TQ may enhance the sensitivity of PC cells to GEM by inhibiting the neurogenic Notch homolog protein 1/phosphatase and tensin homolog (Notch1/PTEN) pathway. A decrease in activated Notch1 and PTEN protein levels can predict patient prognosis and chemotherapeutic resistance. GEM can induce the upregulation of Notch1 in PC cells and increase the expression of the Notch intracellular domain (Zhang et al., 2013). TQ pretreatment significantly reduces the GEM-induced upregulation of Notch1 and Notch intracellular domain; it also restores the PTEN protein level that is downregulated by GEM. The second proposed mechanism involves the phosphoinositide 3 kinase/protein kinase B/mammalian target of rapamycin (PI3K/Akt/mTOR) signaling pathway, which is related to the survival and chemoresistance of PC cells. The activation of this pathway and expression of the downstream effector molecule S6 ribosomal protein can enhance chemosensitivity and promote apoptosis in PC cells However, TQ pretreatment significantly attenuates the phosphorylation of mTOR, S6, and upstream Akt caused by GEM. In the third proposed mechanism, TQ enhances GEM-induced antitumoral activity *in vivo* through nuclear factor kappa-B (NF-κB) and its downstream molecules. NF-κB is one reason for the characteristic resistance of pancreatic tumor cells to the apoptotic effects of chemotherapy drugs. Thus, X-linked inhibitor of apoptosis protein (XIAP) and survivin, which are members of the NF-κB-regulated family of apoptosis inhibitory proteins, are regarded as therapeutic targets because of their involvement in tumorigenesis, especially in chemotherapeutic resistance, cell proliferation, and angiogenesis. Researchers previously found that, compared to GEM administration alone, the DNA-binding activity of NF-κB and structural phosphorylation of p65 is significantly reduced in tumor-bearing mice treated with TQ and GEM. In addition, TQ can significantly affect the downstream molecules of NF-κB by downregulating B cell leukemia/lymphoma-2 (Bcl-2), Bcl-cl, XIAP, and survivin and upregulating the activity of the apoptosis-related caspase-3 and caspase-9. Wang also found that TQ combined with GEM can significantly inhibit the expression of XIAP and MMP-9 in PC tissues, thereby affecting the growth and metastasis of tumors (Wang, 2011). In the fourth proposed mechanism, TQ + GEM can achieve stronger apoptotic efficacy than GEM alone by increasing the arrest of the cell cycle in the G1 phase. In addition, compared to GEM or TQ alone, a combined treatment of TQ and GEM has been found to significantly reduce the tumor weights of tumor-bearing mice without causing a serious increase in toxicity depending on weight loss. A study by Pandita et al. confirmed that compared to GEM alone, the combined application of TQ and GEM reduces the survival rate of PC cells by more than 50%. Similarly, in a PC xenograft model, the combined application of TQ and GEM had a higher efficacy (approximately 80%) in impacting tumor weight. Combined drug administration has also been found to reduce the expression of pyruvate kinase isozyme type M2 (PKM2) in PC cells. PKM2 is the key enzyme of the Warburg effect in PC, which promotes glucose uptake and reduces oxygen consumption, thereby ensuring the growth of PC cells (Pandita et al., 2014). Some researchers have conducted preliminary studies on the relationships between TQ, small molecule RNAs, and GEM and found that the cytotoxic and apoptotic potentials of low-dose GEM suggest a synergistic role in GEM-sensitive and -resistant cell lines transfected with miR-101 and miR-24-2 or treated with co-administered TQ. The synergistic mechanisms of these two microRNAs and TQ in cancer cell lines involve an increase in procaspase3 and PARP expression and a decrease in PKM2 activity (Pandita et al., 2015). Wu et al. also found that TQ can effectively enhance the inhibitory GEM effect on PC cell proliferation, where the synergistic effect mainly involves apoptosis induction (Wu et al., 2014).

Although more in-depth studies on TQ enhancing GEM sensitivity have been published, no prior study has examined whether TQ improving GEM-chemosensitivity of PC is related to collagen. As a phytochemical, TQ showed extraordinary antitumor effects in various cancers. In our study, we found that both TQ and GEM suppress tumor cell migration and invasion and promote apoptosis of PC cells. Moreover, the combination of TQ and GEM was more effective than GEM alone, indicating that the two drugs have a synergistic effect. In addition, we studied the mechanism by which TQ enhances the GEM sensitivity of PC cells. As interstitial fibrosis and hypoxia are important features of PC, interstitial fibrosis in the PC microenvironment is considered the main cause of poor drug delivery efficacy. Our data suggest that TQ may enhance GEM sensitivity by influencing pancreatic interstitial fibrosis. We found that TQ can affect key proteins of ECM production *in vitro* and *in vivo*, thereby affecting tumor cell invasion and GEM sensitivity. In addition, TQ modulated the expression of key proteins of the TGFβ/Smad pathway, suggesting that TQ regulates ECM production through this pathway. HIF-1α plays a key role in the above regulatory mechanisms.

In our study, we proposed that the recently identified and purified component TQ might have potential therapeutic effects in PC, which provides a new direction for research on antitumor drugs that contribute to increased survival time of patients with PC. In addition, our study closely related the characteristics of interstitial fibrosis and hypoxia in PC with low sensitivity to chemotherapy, and found that TQ could improve the sensitivity of PC to chemotherapy by improving interstitial fibrosis for the first time, which provided a certain basis for the study of the relationship between microenvironment and chemotherapy in PC. Less than perfect, our study did not rigorously validate our hypothesis, and may require additional rescue experiments, such as further observation of GEM effects after the removal of interstitial fiber components from PC in animal studies. In addition, since HIF-1α exists only under hypoxic conditions, our experiments were mainly carried out in a low-oxygen environment. Due to financial constraints, some details of the experimental design were imperfect. For example, the intermediate factors of the TGFβ/Smad pathway, Smad2 and Smad3, were only determined regarding the regulation of ECM by TQ, whereas their influence in experiments with combined TQ + GEM administration or HIF-1α overexpression/knockout was not determined and only the key factor of this pathway, TGFβ1, was assessed. However, we are convinced that these limitations did not substantially influence the conclusions of our experiments.

## 5 Conclusion

**FIGURE 15:**
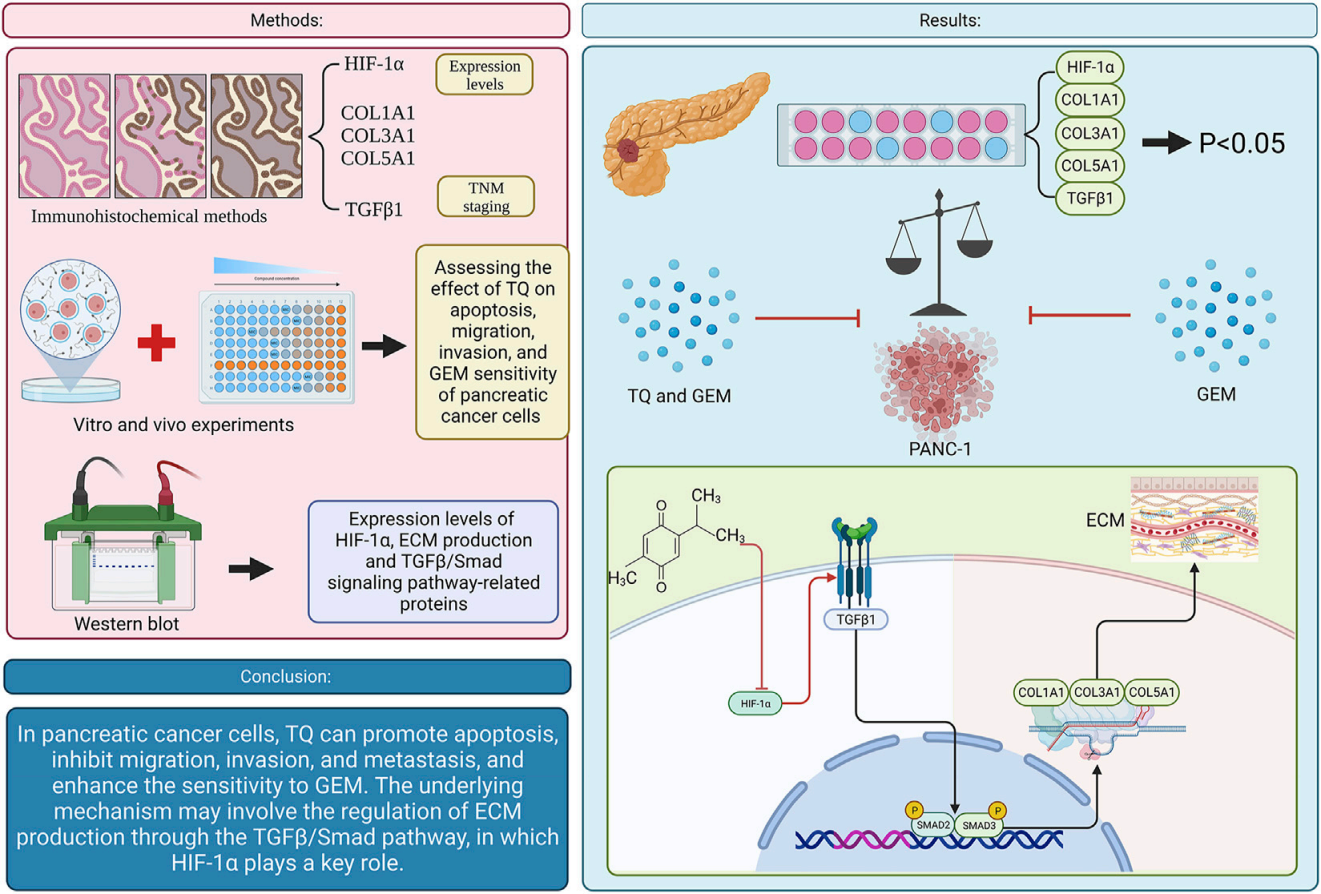
Graphical abstract. (TQ: thymoquinone; GEM: gemcitabine; HIF-1α: hypoxia inducible factor-1α; TGFβ1: transforming growth factor-β1; ECM: extracellular matrix).

In PC, TQ promotes apoptosis, inhibits tumor cell migration, invasion, and metastasis, and enhances the sensitivity of PC cells to GEM. The mechanism by which TQ regulates ECM production involves the TGFβ/Smad pathway, in which HIF-1α plays a key role (Figure 15).

## Data Availability

The original contributions presented in the study are included in the article/Supplementary Materials, further inquiries can be directed to the corresponding author.
